# Urinary neutrophil gelatinase-associated lipocalin in dogs: accuracy of a novel rapid test and biomarker behavior across clinical settings

**DOI:** 10.1093/jvimsj/aalag020

**Published:** 2026-02-17

**Authors:** Filippo Tagliasacchi, Alessia Facchin, Jari Zambarbieri, Marzia Cozzi, Simone Camelliti, Stefania Lauzi, Saverio Paltrinieri, Alessia Giordano, Paola Scarpa

**Affiliations:** Department of Veterinary Medicine and Animal Sciences—DIVAS, University of Milan, Lodi (LO) 26900, Italy; Department of Veterinary Medicine and Animal Sciences—DIVAS, University of Milan, Lodi (LO) 26900, Italy; Department of Veterinary Medicine and Animal Sciences—DIVAS, University of Milan, Lodi (LO) 26900, Italy; PRIMA Lab SA, Via Antonio Monti 7, Balerna-TI 6828, Switzerland; PRIMA Lab SA, Via Antonio Monti 7, Balerna-TI 6828, Switzerland; Department of Veterinary Medicine and Animal Sciences—DIVAS, University of Milan, Lodi (LO) 26900, Italy; Department of Veterinary Medicine and Animal Sciences—DIVAS, University of Milan, Lodi (LO) 26900, Italy; Department of Veterinary Medicine and Animal Sciences—DIVAS, University of Milan, Lodi (LO) 26900, Italy; Department of Veterinary Medicine and Animal Sciences—DIVAS, University of Milan, Lodi (LO) 26900, Italy

**Keywords:** AKI, diagnosis, dog, marker, point-of-care, renal

## Abstract

**Background:**

Urinary neutrophil gelatinase-associated lipocalin (uNGAL) has emerged as an early marker of acute kidney injury (AKI) in dogs, but its measurement by ELISA is laborious in clinical practice.

**Hypothesis/Objectives:**

Assess the performance of a novel point-of-care (POC) uNGAL assay for early detection of AKI in dogs and evaluate the clinical utility of uNGAL in differentiating AKI from other urinary conditions in dogs.

**Animals:**

Urine supernatants from 200 client-owned dogs were collected and grouped as follows: healthy, chronic kidney disease (CKD), AKI (including AKI on CKD), urinary tract infections, urolithiasis, and extrarenal inflammatory diseases. Dogs then were classified by the presence (*n* = 39) or absence (*n* = 161) of AKI for calculation of diagnostic performance.

**Methods:**

Urinary NGAL was measured using the Dog NGAL ELISA Kit (Bioporto) as the existing test and the “PRIMA Veterinary—KI screening test” (PRIMA Lab) as an index test.

**Results:**

At the optimized cut-off of 20 ng/mL for the POC device, a sensitivity of 97.3% (95%CI, 85.8-99.9) and a specificity of 66.3% (95%CI, 58.4-73.5) for diagnosing AKI were found. For the ELISA, a sensitivity of 97.3% (95%CI, 85.8-99.9) and a specificity of 80.4% (95%CI, 73.4-86.2) were found. Cohen’s kappa coefficient (κ = 0.82) indicated an excellent agreement between methods.

**Conclusions and clinical importance:**

With both methods, uNGAL showed moderate specificity and excellent sensitivity for the diagnosis of AKI. The POC device represents a clinically relevant diagnostic tool for screening AKI in patients at risk, given excellent agreement with the existing test.

## Introduction

Neutrophil gelatinase-associated lipocalin (NGAL) is a bacteriostatic molecule that binds to bacterial siderophores, decreasing iron bioavailability.^1^ Circulating NGAL, produced mainly by neutrophils and epithelial tissues, is freely filtered by glomeruli and almost entirely reabsorbed in proximal tubules, making its urinary concentration almost undetectable under physiological conditions.

Increased urinary NGAL (uNGAL) concentration has been observed in humans and dogs during renal parenchymal injury, because of decreased tubular reabsorption and active secretion. Therefore, this molecule has been identified as an early marker of acute kidney injury (AKI).^2-13^

The hallmark of uNGAL is its distinction as an earlier indicator compared to conventional markers of glomerular filtration rate, highlighting its enhanced sensitivity.^5,6^ In dogs, uNGAL outperformed serum creatinine concentration in diagnosing AKI secondary to ischemia and reperfusion injury, with an increase as early as 2 h after the ischemic event and a peak concentration at 12 h.^6^ The increase in uNGAL preceded azotemia in dogs with gentamicin-induced nephrotoxicity, with its decrease indicating the onset of renal recovery. It has high sensitivity for diagnosing grade I AKI secondary to parvoviral enteritis, snake envenomation, babesiosis, heatstroke, and nephrotoxicity.^6-9,14-17^

Local or systemic inflammation has a strong impact on the specificity of uNGAL.^18^ Conditions such as chronic kidney disease (CKD), lower urinary tract inflammation, pyuria, leishmaniasis, myxomatous mitral valve disease (MMVD), and systemic inflammation are reported to cause increases in uNGAL in dogs.^19-28^

Human and canine NGAL can exist as a monomer, dimer and as part of the NGAL/Matrix metalloproteinase-9 complex.^29,30^ Western blot can aid in distinguishing the various forms, differentiating renal and extrarenal diseases. However, the use of uNGAL in veterinary practice is complicated by the laborious measurement. The existing ELISA method requires specialized laboratories and prolonged reporting times, undermining its role as an early biomarker of AKI, a condition that can rapidly progress toward adverse outcomes.

In this context, our primary objective was to evaluate the clinical performance of a point-of-care (POC) test for uNGAL measurement, specifically by calculating its sensitivity and specificity for the diagnosis of AKI. Our secondary aim was to assess the agreement between the POC test and the existing ELISA method, widely applied in research in dogs.^7-12^ Finally, by measuring uNGAL concentration using both tests across a large and diverse cohort of subjects, a further objective was to evaluate findings previously reported in the literature and explore potential new applications in dogs. The hypothesis was that the POC test would demonstrate strong agreement with the existing method of uNGAL quantification, with comparable diagnostic sensitivity and specificity. Such findings would support its value as a reliable screening tool for AKI in dogs.

## Materials and methods

### Study design

**Figure 1:**
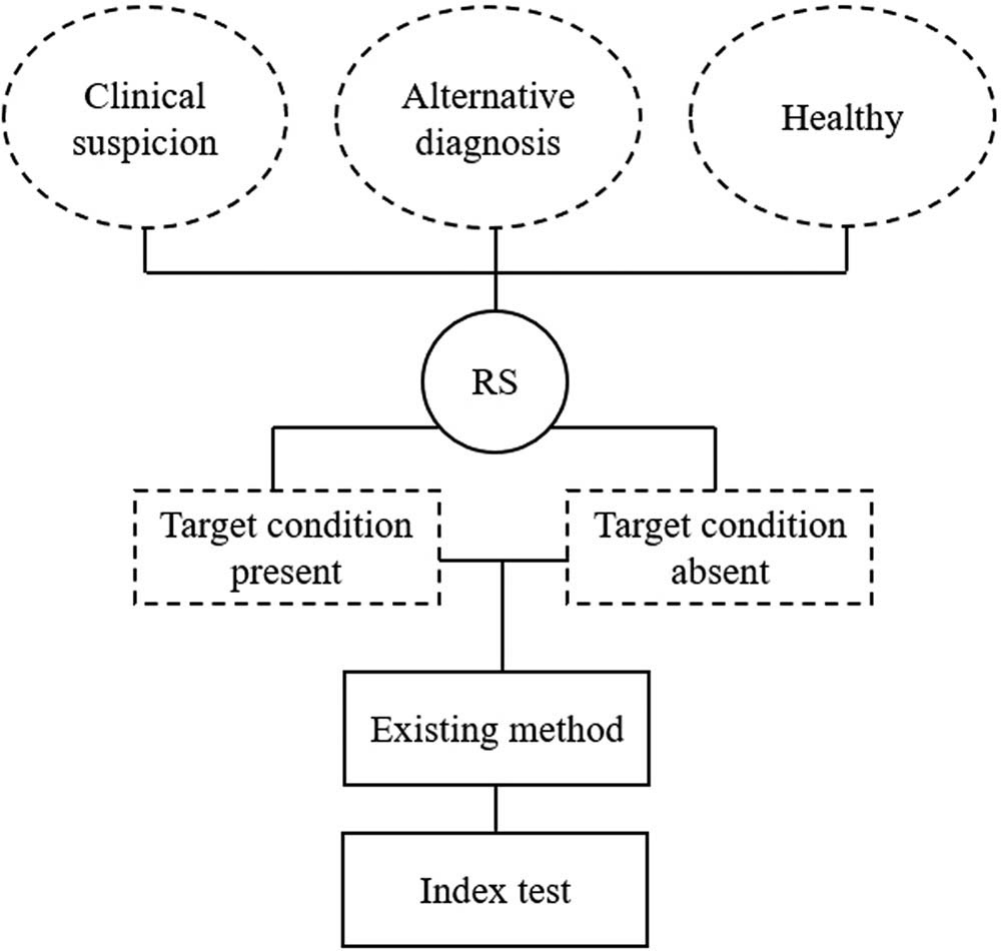
Flow diagram of the fully paired, multiple-gate, case–control diagnostic accuracy study. Multiple gates were considered for patients’ enrollment. All enrolled patients were classified, based on the reference standard, according to the presence or absence of the target condition. Both diagnostic tests were applied to each patient. Abbreviations: RS = reference standard.

A fully paired, multiple-gate, case–control diagnostic accuracy study was conducted on client-owned dogs referred to the Veterinary Teaching Hospital (VTH) of the University of Milan between August 2019 and September 2023 (Figure 1).

### Samples

All dogs referred to the internal medicine service during the study period initially were considered. The source population included dogs that had hematology, serum biochemistry, and urinalysis performed, with an appropriately stored urine volume. Two-hundred client-owned dogs, retrospectively selected from the VTH laboratory database were eligible for group assignment. All patients were > 6 months of age.

All samples were sent in syringes or sterile containers without preservatives to the clinical pathology laboratory. Upon arrival, 5 mL of urine was transferred to a sterile tube and centrifuged at 450 g for 5 min. The remaining supernatant was transferred to 1.5 mL Eppendorf tubes and stored at −80°C. Maximum samples storage time was 3 years. Frozen samples were included in the study when the following inclusion criteria were met: urine samples collected by cystocentesis from the dogs in the study group; no abnormal macroscopic color or turbidity changes; and dipstick analysis and urinary sediment examination available.

Healthy subjects belonged to the hospital staff. Urine samples were collected by spontaneous micturition and included only if urinalysis was normal (dipstick analysis and sediment evaluation).

### Group assignment

Based on history, clinical and laboratory data, dogs were grouped in the following categories: urinary tract infection (UTI), CKD, AKI (including a subgroup of dogs affected by AKI on CKD [ACKD]), extrarenal diseases (divided into inflammatory and noninflammatory), urolithiasis, and healthy dogs.

The diagnoses of CKD and AKI were defined according to International Renal Interest Society (IRIS) guidelines. Inclusion criteria were established in accordance with a previous study.^21^ Patients in the ACKD group met the same classification criteria as AKI dogs, but in the presence of a documented history of CKD and compatible imaging findings. Dogs belonging to the UTI group had compatible clinical signs (eg, dysuria, stranguria, pollakiuria, hematuria), positive urine culture, normal serum creatinine and urea concentrations, and no antibiotic treatment administered. Dogs with urolithiasis had imaging findings compatible with nonobstructive uroliths and normal serum (sCr) creatinine and urea concentrations. Dogs with extrarenal diseases were included only if non-azotemic and with negative urine culture. To be classified in the subgroup of extra-renal inflammatory disease, 2 of the following were required: (1) neutrophilic leukocytosis (neutrophils > 20 × 10^3^/μL), (2) neutropenia with toxic neutrophils (neutrophils < 3 × 10^3^/μL), (3) imaging findings compatible with inflammatory foci, and (4) increased C-reactive protein concentration (> 15 mg/L).

For the healthy group, no abnormalities were identified on physical examination, hematology, serum biochemistry, and urinalysis.

The diagnosis of multiple concurrent urinary conditions was a general exclusion criterion. An aerobic bacterial urine culture was required for the inclusion of subjects affected by UTI, AKI, and extra-renal diseases. For the remaining clinical settings, consistent with previous studies in dogs, an inactive urinary sediment was considered predictive of a negative urine culture result.^31^

### Ethical approval

All samples were from client-owned dogs presented for diagnostic investigations at the University of Milan VTH, during routine health screenings. The owners signed an informed consent form, allowing residual samples, collected as part of the diagnostic procedures, to be used for research purposes. Therefore, according to the statements of the Ethics Committee of the University of Milan (number 2/2016), no further approval by the Institutional Animal Care and Use Committee was necessary.

### Analytical procedures

Frozen urine supernatants were thawed and divided into 2 aliquots, intended for uNGAL measurement by a quantitative existing test “Dog NGAL ELISA Kit” (Bioporto) and semiquantitative immunochromatographic lateral flow POC assay “PRIMA Veterinary—KI screening test” (PRIMA Lab). This test employs specific canine anti-NGAL monoclonal antibodies coated with gold nanoparticles. For the ELISA test, after being vortexed, samples were diluted 1:300 with the diluent buffer provided by the manufacturer, to achieve a concentration of uNGAL within the method linearity (corresponding to 4-400 pg/mL as stated by the manufacturer). The ELISA assay then was performed following the manufacturer’s instructions, and the plate was read at 450 and 620 nm. Optical densities were compared with those of the different calibrators provided with the kit. After being analyzed within a 4-parameter calibration curve, the ELISA results were multiplied by the dilution factor and transformed into nanograms per milliliter. All samples then were analyzed using the POC test, according to the manufacturer’s instructions. Specifically, samples were diluted 1:10 to a final volume of 800 μL and 3 drops were poured into the device. Results were interpreted after 10 min of incubation at room temperature. The color intensity of the positivity bar was compared with the color chart provided by the manufacturer, resulting in the semiquantitative classification of values as negative (0 ng/mL), mildly positive (4 ng/mL, POS 1), moderately positive (20 ng/mL, POS 2), and strongly positive (90 ng/mL, POS 3).

### Data analysis

Statistical analysis was performed using Med Calc 16.1.2 (MedCalc Software bvba) and Analyse-it v 5.66.

Descriptive analyses on data obtained using the Dog NGAL ELISA Kit, established data distribution and variability in the groups and subgroups were performed. The Shapiro–Wilk test was used to evaluate data distribution. Normally distributed data were expressed by mean and SD and non-normally distributed data by median and interquartile range (IQR).

Groups were compared based on the ELISA results. The Kruskal–Wallis test was used to test differences in uNGAL concentrations across different clinical settings, IRIS-AKI grades, and IRIS-CKD stages. A post-hoc Steel–Dwass test was applied to assess uNGAL differences between groups and based on presence or absence of pyuria (considering a threshold of 5 WBC/high-power field [hpf]), hematuria (considering a threshold of 5 RBC/hpf), and urinary tract infection (considering the results of the urine culture). Pearson’s chi-squared analysis was applied on the semiquantitative results of the POC test to evaluate differences in the proportion of positive and negative results among clinical settings, IRIS-AKI grades, and IRIS-CKD stages and to assess differences based on presence or absence of infection, hematuria, and pyuria.

The population was dichotomized according to the presence or absence of AKI. Specifically, the criteria considered as reference standard for defining the presence of AKI were USG < 1.025, and sCr > 1.6 mg/dL in the absence of previous azotemia or an increase in sCr ≥ 0.3 mg/dL within 48 h. Positivity for ≥ 2 of the following criteria also was required: (1) failure to resolve azotemia or return to baseline sCr within 24 h after correction of dehydration and hypovolemia, (2) history of exposure to nephrotoxins, (3) diagnosis of leptospirosis based on positive PCR (blood or urine) or microscopic agglutination titer, and (4) oliguria or anuria despite adequate fluid therapy. Urinary NGAL concentration was compared between the 2 groups. For both methods, results were classified as true positive if the dogs received a diagnosis of AKI (according to the reference standard) and had a value higher than the optimal threshold calculated. Conversely, a true negative result was defined as a dog not diagnosed with AKI, with a value lower than the respective threshold. The diagnostic performance of both methods was calculated using a receiver operating characteristic (ROC) curve and calculating the Youden index.

For the continuous ELISA results, the best-performing cut-off for AKI discrimination was established. For the uNGAL POC device, the best-performing cut-off was selected among those provided.

Patients were dichotomized as “positive” or “negative” for AKI, based on the best uNGAL diagnostic threshold determined as described above. Agreement between the 2 tests was established using Cohen’s kappa coefficient (κ), in 2 steps: first, a contingency table was created to calculate the frequency of correct samples classification with the POC test at the established threshold, compared to the quantitative value defined by the ELISA test. Then, a contingency table was created to assess the concordance between the 2 methods in classifying positive or negative AKI status.

## Results

### Study population

From August 2019 to September 2023, 1231 urine samples from dogs admitted to the internal medicine service of the VTH were selected from the database. The majority of these patients had chronic diseases, for which multiple monitoring samples were available. Consequently, considering only 1 sample per patient, 691 samples were excluded. A total of 217 samples were excluded for comorbidities or incomplete clinical or analytical data necessary for categorization. Finally, 123 samples were excluded because of macroscopic color or turbidity changes, inappropriate sampling method, or insufficient urine volume.

Out of 200 dogs, 100 were males (68 intact and 32 neutered) and 100 were females (32 intact and 68 spayed). The median age was 8 years (IQR = 5-11). There were 64 mixed breed dogs and 136 purebreds.

Out of 200 urine samples, 39 were included in the AKI group (19.5%), of which 15 were classified as ACKD; 38 in the CKD group (19%); 38 in the UTI group (19%); 39 in the healthy group (19.5%); 7 in the urolithiasis group (3.5%); and 39 in the extrarenal disease group (19.5%), of which 16 were classified as noninflammatory (eg, endocrinopathies, gastroenteropathies cardiovascular disease, and neoplasia) and 23 as inflammatory (eg, pneumonia, discospondylitis, and neoplasia). No significant differences in age or sex distribution were observed between the healthy group and the overall population.

### Existing test (Dog NGAL ELISA Kit, Bioporto)

**Figure 2:**
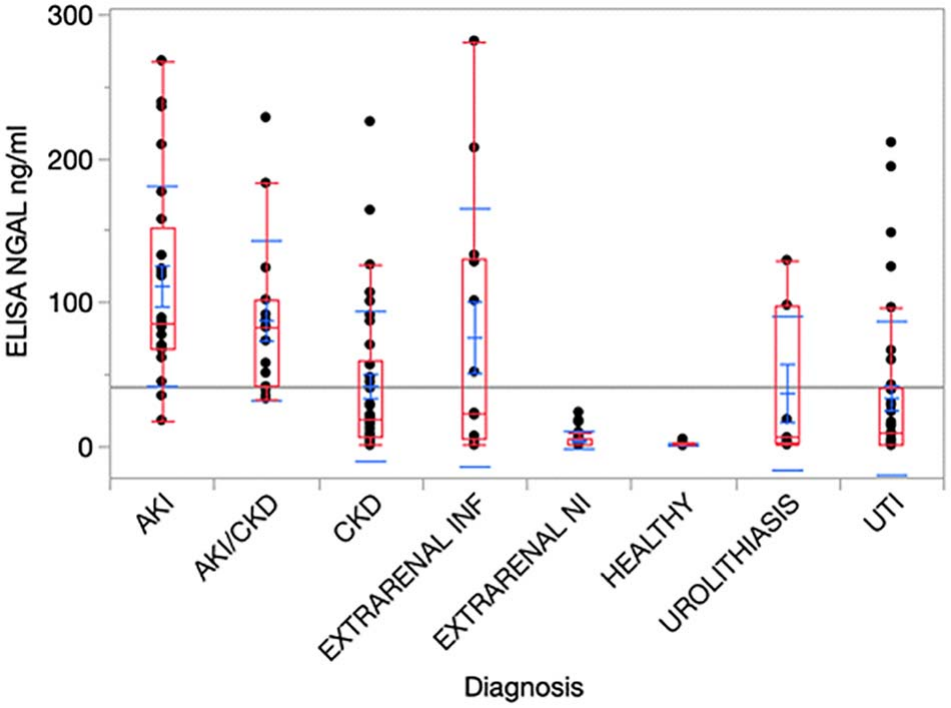
Urinary neutrophil gelatinase-associated lipocalin concentrations in different clinical settings. Each dot represents an individual patient’s result. The bar represents the median, and the whiskers represent the interquartile range. *P* value: *P* < .0001. Abbreviations: ACKD = acute kidney injury on chronic kidney disease; AKI = acute kidney injury; CKD = chronic kidney disease; EXTRARENAL INF = inflammatory extrarenal diseases; EXTRARENAL NI = noninflammatory extrarenal diseases; NGAL = neutrophil gelatinase-associated lipocalin; UTI = urinary tract infection.

Results of the ELISA method in the different clinical settings (groups and subgroups) are listed in Table 1 and summarized in Figure 2. A Kruskal–Wallis test showed significant differences between the groups (*P* < .0001) in terms of uNGAL concentration. The effect size corresponded to *r* = 0.74 indicating a large effect (95%CI, 0.60-0.87). The highest median values were found in dogs with AKI followed by dogs with ACKD (Table 1). The results of the CKD, UTI, and urolithiasis groups were lower, whereas the median concentrations in healthy dogs and those with noninflammatory extrarenal conditions were below the assay range and close to the limit of detection. However, in all groups with medium to high uNGAL concentrations, the scatter of data around the median value was wide (Figure 2). Statistical comparison of individual groups confirmed higher median uNGAL results for the settings AKI and ACKD if compared with dogs affected by CKD (*P* = .0004 and *P* = .02, respectively) or UTI (*P* < .0001 for AKI, *P* = .003 for ACKD). The results of the CKD group were not significantly different from the UTI groups (*P* = .76). Healthy subjects showed median concentrations of uNGAL significantly lower compared to all other groups (*P* < .0001 for the settings AKI, ACKD, CKD, and UTI; *P* = .0002 for extrarenal inflammation; *P* = .02 for urolithiasis), except for the setting of extrarenal noninflammatory diseases (*P* = .48).

**Table 1:** Median values (in bold) and interquartile range of urinary neutrophil gelatinase-associated lipocalin (Dog NGAL ELISA Kit) in different clinical settings, in different grades of acute kidney injury, and in different stages of chronic kidney disease.

**Diagnosis**	***N***	**Median** **(ng/mL)**	**IQR I-III**
**AKI**	14	**85.4**	(67.5-151.4)
**ACKD**	15	**82.4**	(41.2-101.7)
**CKD**	38	**18.8**	(6.4-59.8)
**Extrarenal Inf**	23	**22.8**	(5.3-130.4)
**Extrarenal NI**	16	**1.3**	(0.7-5.6)
**Healthy**	38	**0.9**	(0.6-1.3)
**Urolithiasis**	7	**5.8**	(2.1-97.1)
**UTI**	38	**8.6**	(1.4-39.8)
**IRIS-AKI grade**	***N***	**Median** **(ng/mL)**	**IQR I-III**
**IRIS-AKI 1**	1	**73.2**	(67.5-73.2)
**IRIS-AKI 2**	4	**50.9**	(33.3-149.3)
**IRIS-AKI 3**	13	**84.6**	(69.1-219.1)
**IRIS-AKI 4**	14	**86.8**	(73.2-118.9)
**IRIS-AKI 5**	7	**50.7**	(38.7-123.9)
**IRIS-CKD stage**	***N***	**Median** **(ng/mL)**	**IQR I-III**
**IRIS-CKD 1**	8	**12.1**	(4.5-26.0)
**IRIS-CKD 2**	22	**17.4**	(6.1-54.9)
**IRIS-CKD 3**	3	**29.7**	(6.7-100.5)
**IRIS-CKD 4**	5	**47.8**	(13.5-98.2)

Abbreviations: IQR, interquartile range; *N*, numerosity; AKI, acute kidney injury; ACKD, acute kidney injury on chronic kidney disease; IRIS, International Renal Interest Society; CKD, chronic kidney disease; Extrarenal Inf, extrarenal inflammatory diseases; Extrarenal NI, extrarenal noninflammatory diseases; UTI, urinary tract infection.

The “*N*” column reports the total number of patients on which the data are calculated.

No significant differences were found between the inflammatory group and the AKI (*P* = .63), ACKD (*P* = .93), CKD (*P* = .96), UTI (*P* = .71), and urolithiasis (*P* = .93) groups. Significantly higher median values were found in the inflammatory group compared with healthy subjects and the extrarenal noninflammatory disease group (respectively, *P* = .0002 and *P* = .01). Samples classified as noninflammatory yielded lower median concentrations of uNGAL than all other groups, except for healthy dogs (*P* < .0001 for AKI and ACKD; *P* = .0001 for CKD; *P* = .02 for UTI; and *P* = .02 for urolithiasis). In dogs with urolithiasis, no significantly different concentrations of uNGAL were recorded compared to the AKI (*P* = .21), ACKD (*P* = .49), CKD (*P* = .99), and UTI (*P* = 1) groups. Details on point estimates, measures of uncertainty, and *P* values for all of the comparisons are provided in Table S1.

No significant differences were found in uNGAL concentrations among the different IRIS AKI grades (Table 1), and different IRIS CKD stages (Table 1).

**Figure 3:**
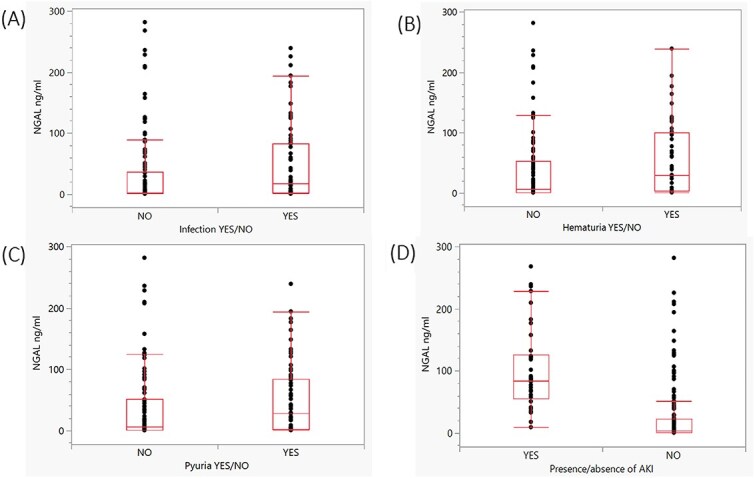
(A) Urinary neutrophil gelatinase-associated lipocalin concentrations in patients with positive or negative urine culture. Each dot represents an individual patient’s result. The bar represents the median, and the whiskers represent the interquartile range. *P* value: *P* = .002. (B) Urinary neutrophil gelatinase-associated lipocalin concentrations in patients with or without hematuria. Each dot represents an individual patient result. The bar represents the median, and the whiskers represent the interquartile range. *P* value: *P* = .01. (C) Urinary neutrophil gelatinase-associated lipocalin concentrations in patients with or without pyuria. Each dot represents an individual patient’s result. The bar represents the median, and the whiskers represent the interquartile range. *P* value: *P* = .01. (D) Urinary neutrophil gelatinase-associated lipocalin concentrations in patients affected or not affected by AKI. Each dot represents an individual patient’s result. The bar represents the median, and the whiskers represent the interquartile range. *P* value: *P* < .0001. Abbreviations: AKI = acute kidney injury; hpf = high-power field; NGAL = neutrophil gelatinase-associated lipocalin; RBC = red blood cell; WBC = white blood cells.

Urine culture-positive samples had significantly higher median concentrations of uNGAL compared with sterile samples (*P* = .002; Figure 3A). The effect size corresponded to *r* = 0.23 indicating a small effect (95%CI, 0.08-0.38). A higher uNGAL concentration was observed in dogs with hematuria (*P* = .01) or pyuria (*P* = .01), compared with dogs without pyuria and hematuria (Figure 3B and C). Effect size corresponded, respectively, to *r* = 0.20 (95%CI, 0.065-0.35) and *r* = 0.21 (95%CI, 0.066-0.36), indicating a small effect. When the samples were dichotomized based on presence (*n* = 39) or absence (*n* = 161) of AKI, a higher median concentration of uNGAL was found in the AKI group compared with the group without AKI (*P* < .0001; Figure 3D). The effect size *r* corresponded to *r* = 0.51 indicating a large effect (95%CI, 0.37-0.65).

**Figure 4:**
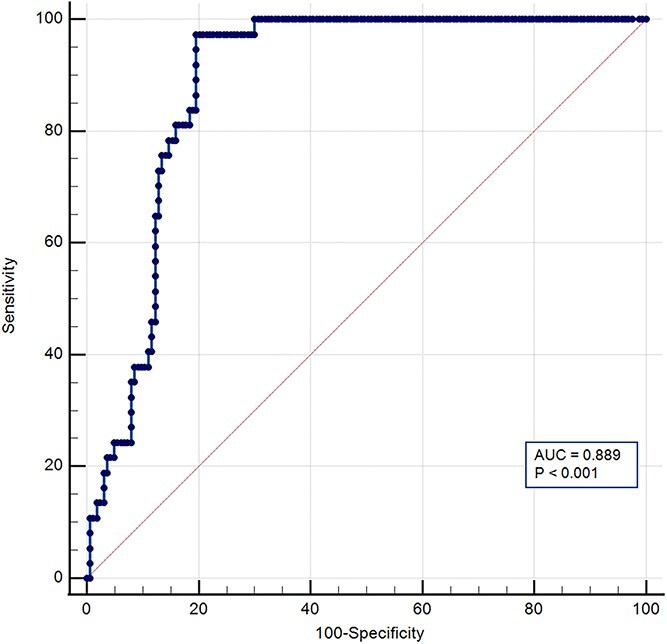
ROC curve analysis for urinary neutrophil gelatinase-associated lipocalin (Dog NGAL ELISA Kit). ROC curve analysis revealed that the optimal cutoff for uNGAL was 29.8 ng/mL, which resulted in a sensitivity of 97.3% and specificity of 80.4% for diagnosis of AKI. The 45-degree diagonal line running from (0,0) to (1,1) represents the line of no discrimination. The AUC was 0.889. Sensitivity is displayed along the y-axis, and 100-specificity along the x-axis. Abbreviations: AKI = acute kidney injury; AUC = area under the curve; uNGAL = urinary neutrophil gelatinase-associated lipocalin; ROC = receiver operating characteristic curve.

The ROC curve designed using ELISA results (Figure 4) indicated excellent discrimination of uNGAL for AKI patients (*P* < .0001) from all the other clinical groups considered. The area under the ROC curve (AUC) was 0.889 (95%CI, 0.84-0.93). The highest Youden index (0.777; 95%CI, 0.67-0.83), indicated that the optimal cut-off for the ELISA was 29.8 ng/mL. This cut-off was characterized by a sensitivity of 97.3% (95%CI, 85.8-99.9) and a specificity of 80.4% (95%CI, 73.4-86.2). The positive likelihood ratio was 4.96.

### POC test (PRIMA veterinary—KI screening test, PRIMA Lab)

The results recorded in the different groups and subgroups of dogs with the NGAL POC test are reported in Table 2. Out of the 200 samples, 75/200 were negative, 34/200 mildly positive, 44/200 moderately positive, and 47/200 strongly positive. Pearson’s chi-squared analysis identified a significant difference in the proportion of negative and positive results among the different groups (*P* < .0001). The effect size corresponded to *r* = 0.80 indicating a large effect (95%CI, 0.65-0.95).

**Table 2:** Distribution of negative and positive results of urinary neutrophil gelatinase-associated lipocalin (PRIMA Lab KI screening test) in the different clinical settings.

	**POC test results**				
**True diagnosis**	**NEG**	**POS 1**	**POS 2**	**POS 3**	**Total**
**AKI**	0	1	7	16	24
**ACKD**	0	0	6	9	15
**CKD**	5	10	15	8	38
**Extrarenal Inf**	12	6	3	2	23
**Extrarenal NI**	7	4	2	3	16
**Healthy**	35	4	0	0	39
**Urolyth**	2	3	0	2	7
**UTI**	14	6	11	7	38
**Total**	75	34	44	47	200

Abbreviations: AKI, acute kidney injury; ACKD, acute kidney injury on chronic kidney disease; CKD, chronic kidney disease; Extrarenal Inf, extrarenal inflammatory diseases; Extrarenal NI, extrarenal noninflammatory diseases; NEG, negative; POC, point-of-care; POS 1, mild positivity; POS 2, moderate positivity; POS 3, strong positivity; uNGAL, urinary neutrophil gelatinase-associated lipocalin; UTI, urinary tract infection.

The “NEG” column reports the number of patients with negative results (uNGAL approximately 0 ng/mL). In the “POS1,” “POS 2,” and “POS 3” columns are reported, respectively, the number of patients with a mildly positive (uNGAL approximately 4 ng/mL), moderately positive (uNGAL approximately 20 ng/mL), and strongly positive result (uNGAL approximately 90 ng/mL).

Among the healthy dogs, 35/39 tested negative (89.7%), whereas the remaining 4 showed mild positivity (10.2%). In contrast, moderately to strongly positive results were observed in 23/24 dogs with AKI (95.8%) and in 100% of ACKD subjects. In these groups, no negative results were found and only 1 mildly positive result (0.04%) was found in the AKI group.

Considering the IRIS AKI grades and the IRIS CKD stages, no significant differences were found in the proportion of negative and positive results.

No significant difference was found in the proportion of negative or positive results based on presence or absence of infection (*P* = .12; effect size of *r* = 0.18, 95%CI, 0.035-0.321), pyuria (*P* = .15; effect size of *r* = 0.18, 95%CI, 0.028-0.322), and hematuria (*P* = .09; effect size of *r* = 0.20; 95%CI, 0.045-0.337).

When results were dichotomized based on the presence or absence of AKI (Table 3), 75/161 dogs without AKI (46.6%) tested negative, whereas mild positivity was observed in 33 dogs (20.4%). However, moderate (19.2%) and strongly (13.6%) positive results also were detected (31/161 and 22/161, respectively). In the AKI group, 25/39 dogs (64.1%) had strong positivity, 13/39 dogs (33.3%) had moderate positivity, only 1 dog (0.02%) showed mild positivity, and no negative results were observed. Overall, moderate or strong positivity was recorded in 97.4% of cases. The difference in the proportion of negative and positive results between patients with or without AKI was significant (*P* < .0001). The effect size corresponded to *r* = 0.53 indicating a large effect (95%CI, 0.38-0.68).

**Table 3:** Distribution of negative and positive results of urinary neutrophil gelatinase-associated lipocalin (PRIMA Lab KI screening test) in patients affected or not affected by AKI.

	**POC test results**				
**True diagnosis**	**NEG**	**POS 1**	**POS 2**	**POS 3**	**Total**
**AKI**	0	1	13	25	39
**Without AKI**	75	33	31	22	161
**Total**	75	34	44	47	200

Abbreviations: AKI, acute kidney injury; NEG, negative; POC, point-of-care; POS 1, mild positivity; POS 2, moderate positivity; POS 3, strong positivity; uNGAL, urinary neutrophil gelatinase-associated lipocalin.

The “NEG” column reports the number of patients with a negative result (uNGAL approximately 0 ng/mL). In the “POS1,” “POS 2,” and “POS 3” columns is reported, respectively, the number of patients that obtained a mildly positive (uNGAL approximately 4 ng/mL), moderately positive (uNGAL approximately 20 ng/mL), and strongly positive result (uNGAL approximately 90 ng/mL).

Sensitivity and specificity in discriminating between AKI positive and negative patients were calculated for each cut-off of the POC test (Table 4). The highest Youden index of 0.64 (95%CI, 0.53-0.70) suggested the moderate band of positivity (uNGAL concentration = 20 ng/mL) as the best performing cut-off, with a sensitivity of 97.3% (95%CI, 85.8-99.9) and a specificity of 66.3% (95%CI, 58.4-73.5). The positive likelihood ratio was 2.88. According to ROC curve analysis, the cut-off of 90 ng/mL maximizes specificity, with a sensitivity of 64.9% (95%CI, 47.5-79.8) and a specificity of 88.9% (95%CI, 79.6-90.8).

**Table 4:** Diagnostic performances calculated for each cut-off of the semiquantitative PRIMA Lab KI screening test.

**Cut-off** **(ng/mL)**	**Sensitivity**	**Specificity**	**Likelihood ratio (+)**	**Likelihood ratio (−)**	**Predictive value (+)**	**Predictive value (−)**	**Youden's index**
**4**	1.000	0.460	1.85	0.00	0.30	1.00	0.460
**20**	0.973	0.663	2.88	0.04	0.40	0.99	0.636
**90**	0.649	0.859	4.60	0.41	0.51	0.92	0.508

**Figure 5:**
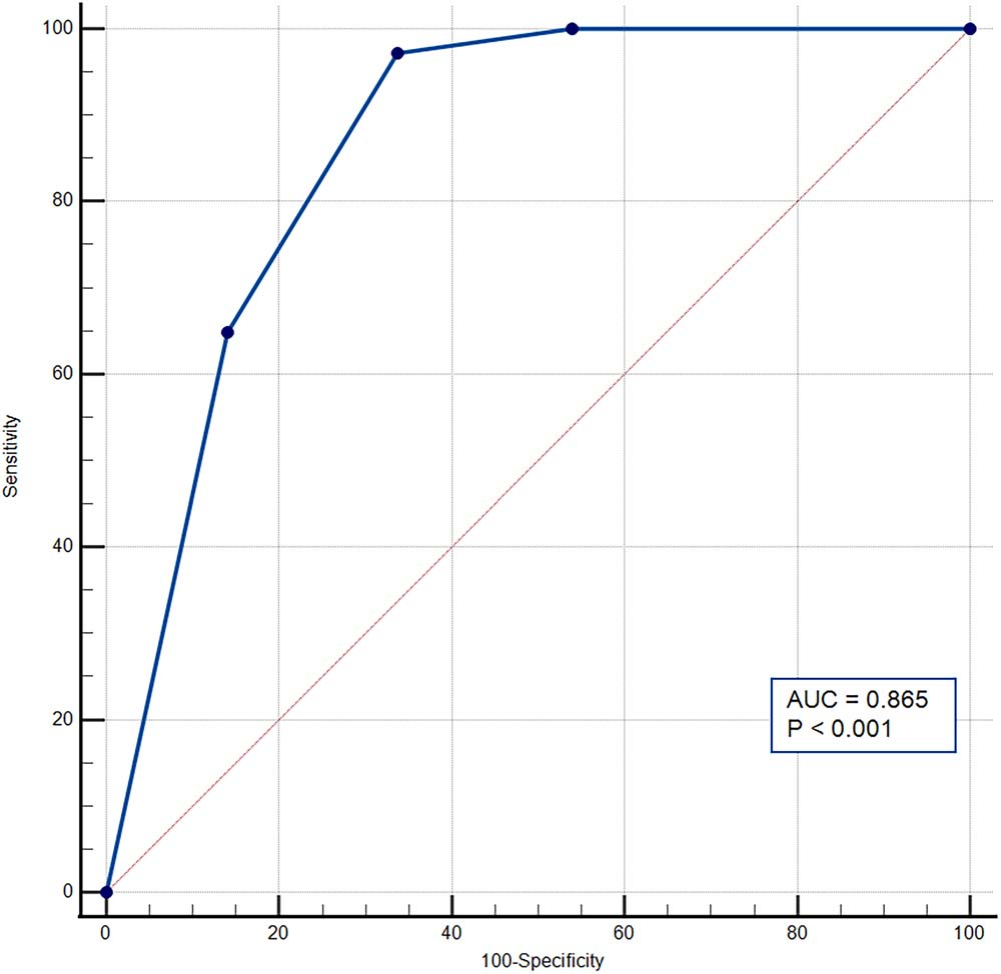
ROC curve analysis for urinary neutrophil gelatinase-associated lipocalin (PRIMA Lab KI screening test). ROC curve analysis indicated that the optimal cutoff for uNGAL, between those given by the manufacturer, was 20 ng/mL, which resulted in a sensitivity of 97.3% and specificity of 66.3% for diagnosis of AKI. The 45-degree diagonal line running from (0,0) to (1,1) represents the line of no discrimination. The AUC was 0.865. Sensitivity is displayed along the y-axis, and 100-specificity along the x-axis. Abbreviations: AKI = acute kidney injury; AUC = area under the curve; uNGAL = urinary neutrophil gelatinase-associated lipocalin; ROC = receiver operating characteristic curve.

The ROC curve designed using the values of the color chart scale (Figure 5) showed an excellent discriminating power of uNGAL for AKI patients (*P* < .0001). The AUC was 0.865 (95%CI, 0.818-0.912).

### Agreement between methods

Results regarding agreement between the methods for the quantification of uNGAL are presented in a contingency table (Table 5). Specifically, 5/80 samples (6.2%) that, according to the existing test had < 4 ng/mL of uNGAL, had weak bands (>4 ng/mL) with the POC test, 11/39 samples (28.2%) that had an ELISA result between 4 and 20 ng/mL had bands corresponding to > 20 ng/mL with the POC test, and 9/43 samples (20.9%) with an ELISA result between 20 and 90 ng/mL had strong positive bands (>90 ng/mL) with the POC test. In this category, only 1 sample with an ELISA result between 20 and 90 ng/mL had a lower result with the POC test. The overall agreement between the 2 methods was classified as excellent (κ = 0.821; 95%CI, 0.758-0.884).

**Table 5:** Agreement between Dog NGAL ELISA Kit (Bioporto) and KI screening test (PRIMA Lab) in classifying patients for each semiquantitative interval of urinary neutrophil gelatinase-associated lipocalin concentration.

	**KI screening test**				
**Dog NGAL ELISA Kit**	**0-4 ng/mL**	**4-20 ng/mL**	**20-90 ng/mL**	**≥90 ng/mL**	**Total**
**0-4 ng/mL**	75	5	0	0	80
**4-20 ng/mL**	0	28	11	0	39
**20-90 ng/mL**	0	1	33	9	43
**≥90 ng/mL**	0	0	0	38	38
**Total**	75	34	44	47	200

Abbreviations: NGAL, neutrophil gelatinase-associated lipocalin.

In this contingency table, each column expressed the number of patients classified by the rapid test as within the specific diagnostic interval. In each row, the number of patients classified by the existing test as within the specific diagnostic interval is expressed. Each semiquantitative interval is established based on the diagnostic threshold given by the manufacturer of the rapid test (PRIMA Lab KI screening test).

The concordance of the 2 methods in classifying patients as affected by AKI using the diagnostic thresholds defined by the ROC curves described above (29.8 ng/mL for the existing test, 20 ng/mL or moderate band for the POC test) also was excellent (κ = 0.784; 95%CI, 0.699-0.869). As shown in Table 6, the most frequent discrepancy was overestimation of positive samples with the POC test, with 21/130 cases (16.1%) classified as potentially affected by AKI that were not likely affected by AKI with the ELISA method. All of the patients possibly affected by AKI according to the ELISA method were correctly classified by the POC test, without false negative results.

**Table 6:** Agreement between Dog NGAL ELISA Kit (Bioporto) and KI screening test (PRIMA Lab) in classifying patients as positive or negative for the diagnosis of acute kidney injury, based on the optimal cut-off calculated.

	**KI screening test**		
**Dog NGAL ELISA Kit**	**NEG**	**POS**	**Total**
**NEG**	109	21	130
**POS**	0	70	70
**Total**	109	91	200

Abbreviations: NGAL, neutrophil gelatinase-associated lipocalin; NEG, negative; POS, positive.

In this contingency table, in each column is expressed the number of patients classified as positive or negative by the rapid test based on the optimal urinary NGAL cut-off of 20 ng/mL. Each row expressed the number of patients classified as positive or negative by the existing test based on the optimal cut-off of 29.7 ng/mL.

## Discussion

We demonstrated that uNGAL concentrations measured using both the existing method (Dog NGAL ELISA Kit, Bioporto) and the POC test (PRIMA Veterinary—KI screening test, PRIMA Lab) effectively discriminated dogs affected by AKI from those without AKI. Both methods achieved excellent diagnostic accuracy, high sensitivity and moderate specificity in detecting AKI. A strong concordance was observed between the 2 methods, confirming the reliability of the POC device in clinical practice. These data are essential to assure the clinical application of this marker as a screening tool for dogs with AKI.

The diagnostic performance of the POC test, at the ideal cut-off, showed sensitivity and specificity similar to those obtained with the ELISA-based NGAL assay. Although mild positivity on the POC test (corresponding to the cut-off of 4 ng/mL) may suggest incipient AKI, albeit with low probability, the 20 ng/mL cut-off optimizes diagnostic performance and agreement with the ELISA method for AKI diagnosis.

Both methods demonstrated excellent sensitivity. Therefore, a negative result on the POC test provides a high level of confidence in ruling out AKI, supporting its use primarily as an exclusionary rather than an inclusionary test.

A bedside test for real-time renal injury detection offers a substantial clinical advantage. Indeed, the clinical challenge in AKI recognition lies in the delayed increases of conventional markers, such as sCr, which may not change during early or mild (IRIS AKI grade 1) injury. This delay narrows the therapeutic window, because interventions are most effective before overt functional decline becomes evident. The early identification of patients at risk for AKI may prompt clinicians to intensify monitoring, optimize hemodynamic support, or modify treatment strategies before irreversible damage occurs.

However, at the ideal cut-off, both methods had impaired specificity, possibly explained by the overlap of uNGAL concentration with other clinical settings, such as extrarenal inflammatory diseases, UTI and CKD. Specifically, 32.9% and 19.8% of patients without AKI showed uNGAL concentrations above the optimal cut-off with the POC test and the conventional method, respectively. This aspect must be taken into consideration when extending the use of the POC test to general practice. A positive test result may reflect the presence of a comorbidity prognostically less relevant than a true diagnosis of AKI. Decreased specificity and the overlapping results between dogs with AKI and CKD represent limitations already described for other markers of tubular injury.^32-35^ From the perspective of using the POC test, such a result should prompt further investigation of suspected AKI using appropriate patient monitoring.

It is important to emphasize that different thresholds can help optimize either sensitivity or specificity, depending on the goals of the clinician. Finally, the balance of sensitivity and specificity could vary depending on the specific target population. In our study, maximizing the specificity of the POC test by adopting the higher cut-off of 90 ng/mL resulted in a marked decrease in sensitivity, thereby limiting its utility as a screening tool. However, the target population for this test is likely to include all patients at risk of, or suspected to have, AKI, with pretest probability influencing test performance accordingly. In contrast, our study population reflected a heterogeneous range of clinical conditions, not necessarily associated with a risk or suspicion of AKI.

Taking into consideration the above-mentioned specificity limitation of the biomarker, it was deemed essential to verify concordance between the POC test and the ELISA method in the quantification of uNGAL, which was excellent with a slight tendency to overestimate.

These results emphasize the potential role of the POC test as a practical tool for early AKI surveillance. Although limited specificity requires cautious interpretation, the ability to rapidly and sensitively identify injury at the patient’s side provides clinicians with actionable information at a stage when intervention is most likely to improve outcomes. In agreement with a previous study, the role of uNGAL is confirmed as a potentially sensitive, and noninvasive biomarker for AKI identification.^9^

Using the ELISA method, higher concentrations of uNGAL are identified in the AKI and ACKD groups than in all other settings, except for the extrarenal inflammatory disease group. A previous study proposed how, during severe systemic inflammation, massive neutrophil recruitment could lead to increased serum and urinary NGAL concentrations, even in the absence of renal injury.^18^ Moreover, patients with severe systemic inflammation are at risk for systemic inflammatory response syndrome, which can rapidly progress to multiorgan dysfunction. Increases in uNGAL in some of these patients, where the clinical course is often peracute and life-threatening, may reflect early AKI, preceding increases in classical markers of GFR.

Given the neutrophilic origin of NGAL, local inflammation can impact its specificity. Lower urinary tract inflammation, CKD, and proteinuria secondary to leishmaniasis are other reported causes of increased uNGAL in dogs.^19,23,28^ Moreover, inflammatory AKI is associated with higher uNGAL concentrations than noninflammatory AKI.^7^ Our findings confirm that uNGAL specificity as a marker of AKI is substantially affected by local and systemic inflammation. Regardless of the diagnostic method, this factor is a major limitation and must be acknowledged when using uNGAL as a screening marker.

A possible role of uNGAL in differentiating renal injury from other urologic conditions was evident in our study. A significant difference between AKI and CKD patients was reported in previous studies.^19,21^

At the same time, median concentrations of uNGAL were significantly higher in the AKI and ACKD groups compared with UTI patients. Such a difference has not been reported previously in dogs. Moreover, the UTI group had significantly higher concentrations of uNGAL compared with healthy subjects and those with noninflammatory extrarenal diseases. This finding is consistent with a previous study.^24^

In the context of antimicrobial stewardship and considering the diagnostic and therapeutic challenges associated with UTI in dogs,^36^^,^^37^ our findings suggest that uNGAL may represent a biomarker of potential interest in this setting. This application has long been explored in human medicine, particularly in pediatrics, where uNGAL has been extensively studied as a marker of UTI. More recently, it has also emerged as a promising method to differentiate UTIs from asymptomatic bacteriuria in elderly women.^38^ In our study, the higher median uNGAL concentrations observed in dogs with positive urine cultures further support the plausibility of these applications. However, the potential diagnostic role of uNGAL to support a suspicion of UTI in dogs or distinguish it from subclinical bacteriuria requires validation by prospective studies.

Consistent with a previous study, no differences in uNGAL concentration were identified among different IRIS AKI stages, with both methods.^7^ Limited numbers of patients in some IRIS AKI groups may have increased individual variability, affecting statistical power. However, another possible explanation lies in the nature of the marker itself. Being an index of tubular damage, uNGAL does not necessarily correlate with sCr, a marker that reflects GFR. The current IRIS AKI classification is based on parameters of functionality, not injury. Moreover, as previously reported in other studies^6^, azotemia is delayed compared with changes in uNGAL. Marked tubular damage therefore could occur even in early IRIS AKI grades, without resulting in an increase in sCr.

Similarly, uNGAL results were not different among IRIS CKD stages using both methods. The possible prognostic value of uNGAL in CKD is promising, as previously reported*.*^12,39^ As a general consideration, very few dogs classified as healthy or IRIS CKD stage 1 had high concentrations of uNGAL. In contrast, uNGAL concentration tended to increase in more advanced stages. Although not significant, this trend potentially could be confirmed with a larger sample.

One limitation of our study is its retrospective nature. Multiple-gate diagnostic accuracy studies are susceptible to spectrum bias. The inclusion of patients with advanced AKI stages may have led to an overestimation of sensitivity. Conversely, including patients with alternative diagnoses yielding uNGAL results compatible with AKI may have decreased specificity. Multiple hypothesis testing was performed, which increases the risk of false-positive findings because of family-wise error. Moreover, the thresholds used for the POC test were derived from the same dataset on which sensitivity and specificity were estimated, which may lead to overly optimistic estimates.

Despite a rigorous approach to patient classification into clinical settings, inaccurate allocation cannot be entirely ruled out. It cannot be excluded that subclinical IRIS AKI grade 1 was present in some patients with systemic inflammation. The identification of individuals in this category is a key goal of current research on uNGAL and requires periodic clinical and laboratory monitoring.

Systemic inflammation often coexists in patients affected by AKI. Distinguishing inflammatory AKI from AKI with concurrent systemic inflammation is challenging in retrospective analyses. This limitation is less impactful when the POC test is used to screen patients at risk of AKI. A positive result should be interpreted as the starting point for a comprehensive diagnostic and monitoring approach.

The categories of extrarenal diseases included a wide range of disorders. Patients were recruited to quantify the impact of extra-renal diseases on uNGAL’s diagnostic performance for AKI. Doing so is important given the growing diagnostic applications of NGAL in nonrenal conditions.^40-42^ Recent evidence about uNGAL in dogs with different stages of MMVD or mammary carcinoma was not considered in the stratification of our patients, thus not excluding their concomitant presence in some of them.^40,41^

### Conclusion

The PRIMA Veterinary—KI screening test (PRIMA Lab) and the existing Dog NGAL ELISA Kit (Bioporto) demonstrated good diagnostic accuracy in detecting AKI in dogs, with excellent sensitivity and moderate specificity. Excellent agreement between the 2 methods confirms the analytical reliability of the POC device. Its primary advantage is to provide rapid, cage-side assessment of tubular injury, facilitating early identification of dogs at risk of AKI, before conventional functional markers become abnormal. Its excellent sensitivity makes it an effective screening test, enabling timely clinical intervention. Although the moderate specificity and overlap with other inflammatory or chronic renal conditions require cautious interpretation, the POC test offers a practical and accessible screening option for AKI.

## Supplementary Material

aalag020_Supplementary_Table_1_clean

## Off-label antimicrobial declaration

The authors declare no off-label use of antimicrobials.

## Institutional animal care and use committee or other approval declaration

Informed consent from the owners, allowing residual samples, collected as part of the diagnostic procedures, to be used for research purposes. Based on the statement of the Ethics Committee of the University of Milan (number 2/2016), no further approval steps by the Institutional Animal Care and Use Committee were required.

## Abbreviations

ACKD: acute kidney injury on chronic kidney disease
AKI: acute kidney injury
AUC: area under the curve
CKD: chronic kidney disease
GFR: glomerular filtration rate
IQR: interquartile range
IRIS: International Renal Interest Society
K: Cohen’s kappa coefficient
MMVD: myxomatous mitral valve disease
NGAL: neutrophil gelatinase-associated lipocalin
POC: point-of care
RBC/hpf: red blood cell per high-power field
ROC: receiver operating characteristic
uNGAL: urinary neutrophil gelatinase-associated lipocalin
UTI: urinary tract infection
WBC/hpf: white blood cell per high-power field
