# Supplementary material for: Urinary neutrophil gelatinase-associated lipocalin in dogs: accuracy of a novel rapid test and biomarker behavior across clinical settings
Source: J Vet Intern Med. 2026 Feb 17;40(1):aalag020. doi: 10.1093/jvimsj/aalag020 (PMC12910616; doi:10.1093/jvimsj/aalag020)
Supplement: aalag020_Supplementary_Table_1_clean [file aalag020_supplementary_table_1_clean.docx]

| **Clinical setting** | **Clinical setting** | **p-value** | **Hodges-Lehmann (ng/mL)** | **LCL** | **UCL** |
| --- | --- | --- | --- | --- | --- |
| UTI | HEALTHY | <.0001* | 7.66 | 0.82 | 27.75 |
| UROLITH | HEALTHY | 0.0194* | 4.97 | 0.16 | 127.04 |
| UTI | EXTRARENAL NI | 0.0189* | 4.47 | 0.15 | 28.07 |
| UROLITH | EXTRARENAL NI | 0.39 | 3.88 | -4.28 | 121.96 |
| EXTRARENAL INF | CKD | 0.97 | 6.63 | -25.50 | 111.59 |
| UTI | UROLITH | 1.00 | -0.71 | -96.70 | 59.06 |
| AKI/CKD | AKI | 0.99 | -16.11 | -87.97 | 42.85 |
| UROLITH | EXTRARENAL INF | 0.93 | -16.98 | -206.78 | 107.02 |
| EXTRARENAL INF | AKI/CKD | 0.93 | -31.87 | -90.80 | 93.26 |
| UROLITH | CKD | 0.99 | -4.63 | -86.01 | 91.32 |
| UROLITH | AKI/CKD | 0.50 | -51.74 | -176.97 | 59.20 |
| EXTRARENAL INF | AKI | 0.73 | -44.13 | -119.51 | 59.14 |
| UTI | EXTRARENAL INF | 0.72 | -18.68 | -123.34 | 15.05 |
| UTI | CKD | 0.75 | -5.37 | -26.74 | 8.44 |
| UROLITH | AKI | 0.25 | -67.04 | -203.82 | 40.02 |
| HEALTHY | EXTRARENAL NI | 0.48 | -0.41 | -2.25 | 0.16 |
| EXTRARENAL NI | EXTRARENAL INF | 0.0099* | -21.62 | -131.41 | -0.91 |
| CKD | AKI/CKD | 0.0212* | -43.51 | -83.40 | -5.10 |
| UTI | AKI/CKD | 0.0031* | -53.22 | -89.17 | -22.12 |
| CKD | AKI | 0.0013* | -62.30 | -113.25 | -17.75 |
| EXTRARENAL NI | AKI/CKD | <.0001* | -74.36 | -101.13 | -38.02 |
| EXTRARENAL NI | CKD | 0.0001* | -15.95 | -46.42 | -4.49 |
| HEALTHY | EXTRARENAL INF | 0.0002* | -21.95 | -131.37 | -3.04 |
| UTI | AKI | <.0001* | -67.99 | -118.70 | -30.48 |
| EXTRARENAL NI | AKI | <.0001* | -83.29 | -139.87 | -59.63 |
| HEALTHY | AKI/CKD | <.0001* | -81.48 | -100.87 | -40.35 |
| HEALTHY | AKI | <.0001* | -84.16 | -131.77 | -66.06 |
| HEALTHY | CKD | <.0001* | -17.68 | -43.55 | -6.30 |

**Supplementary Table 1: Multiple comparisons among clinical settings for median urinary neutrophil gelatinase-associated lipocalin (uNGAL) concentrations, performed using the Steel–Dwass test. Uncertainty estimates are expressed as Hodges–Lehmann differences with corresponding lower and upper confidence limits. The Hodges–Lehmann estimate represents the median difference between groups.** Abbreviations: AKI = Acute kidney injury; ACKD = Acute kidney injury on chronic kidney disease; CKD = Chronic kidney disease; EXTRARENAL INF = Extrarenal inflammatory diseases: EXTRARENAL NI = Extrarenal non-inflammatory diseases; UROLITH= Urolithiasis; UTI = Urinary tract infection; LCL= Lower Confidence Limit; UCL= Upper Confidence Limit.
